# The decision to reimage following extravasation in diagnostic nuclear medicine

**DOI:** 10.3389/fnume.2023.1171918

**Published:** 2023-04-21

**Authors:** Jackson W. Kiser

**Affiliations:** Department of Molecular Imaging, Carilion Clinic, Roanoke, VA, United States

**Keywords:** diagnostic imaging, nuclear medicine, extravasation, PET/CT, quality, patient communication

## Abstract

The primary goal of diagnostic nuclear medicine is to provide complete and accurate reports without equivocation or disclaimers. If specific clinical questions cannot be answered because of radiopharmaceutical extravasation, the imaging study may have to be repeated. The decision to reimage is based on several factors including the diagnostic quality of the images, additional patient radiation dose, patient burden, and administrative constraints. Through process improvement efforts, nuclear medicine departments can significantly reduce the frequency of extravasation and thereby also the need for reimaging. Communication with the patient is important any time extravasation may impact their immediate or future care. The circumstances and potential ramifications should be explained, and patient concerns should be addressed. Although recent arguments have been made in favor of investigating and addressing only those extravasations which result in serious patient injury, patients and their referring physicians deserve to know any time their nuclear medicine study may have been impacted.

## 1. Introduction

Diagnostic nuclear medicine involves the use of radiopharmaceuticals to assess and diagnose conditions such as cancer, infection, and heart disease. Most procedures begin by intravenously administering a radiopharmaceutical—typically into the basilic or cephalic veins in the antecubital fossa, forearm, or hand. Within seconds, the injected material travels through the circulatory system and disperses throughout the body. Specific radiopharmaceuticals are tailored to target specific biological processes within the body (e.g., Fluorodeoxyglucose and metabolism), and concentrate within regions of the body accordingly. Nuclear imaging can then be used to create images from these areas of high and low radiopharmaceutical uptake.

Occasionally, radiopharmaceuticals are accidentally injected partially or entirely into the tissue surrounding the vein, rather than the vein as prescribed. This is known as extravasation and can happen for a variety of reasons, such as, due to misplacement of the needle or structural failure of the vein during injection of the radiopharmaceutical or saline flush (1). The frequency of extravasation within diagnostic nuclear medicine imaging is reported to be 15% with potentially large variability due to center- and individual-specific factors (2–8). During an extravasation, a portion of the injected radiopharmaceutical will infiltrate the tissue instead of entering systemic circulation as intended. Over time, the extravasated material will disperse and clear through the patient's lymphatic system, but those processes take time. There are two ways that an extravasation can impact the patient or their medical care. First, by unanticipated irradiation of injection site tissue, and secondly, by altering the dynamics of radiopharmaceutical biodistribution and uptake and thus compromising the imaging procedure.

When properly administered, radiopharmaceuticals are dispersed throughout the body and result in very low radiation dose for the patient. However, for cases of extravasation, dose to the injection site tissue can be substantial (9–16). The published literature includes examples where radiopharmaceutical extravasation has caused tissue damage such as erythema, desquamation, and necrosis (13, 14, 17–21) as well as increased risk of cancer (22). Harm may take weeks or months to manifest (23–25), though, so prompt identification of extravasation is important to mitigate potential effects.

Extravasations can also impact the patient by delaying systemic circulation of a portion of the injected radiopharmaceutical. Interpretation and quantification of nuclear medicine imaging assumes that the radiopharmaceutical is injected as a bolus and promptly distributed. The amount of time between injection and imaging is chosen to allow for not only organ or tumor uptake of the radiopharmaceutical, but also clearance of it from the patient's vascular system. However, in cases of extravasation, the radiopharmaceutical is reabsorbed by the lymphatic system and deposited into the venous blood over the course of several minutes to hours (9, 26). The concentration of radiopharmaceutical in the blood is elevated at the time of imaging which reduces target-to-background contrast. This can confound identification and assessment of smaller or less avid targets (27).

Additionally, when systemic availability of the radiopharmaceutical is delayed due to extravasation, organ or tumor uptake will be suboptimal at the time of imaging. Because of the decreased uptake, quantitative and semi-quantitative calculations will be inaccurate. One such semi-quantitative measure of uptake is called the Standardized Uptake Value (SUV) and is calculated relative to the injected activity per unit weight of the patient (28). When a portion of the injected radiopharmaceutical is unavailable for uptake, SUV is inaccurate by definition. Other imaging procedures and their respective calculations are also impacted by extravasation. For example, in myocardial perfusion imaging, extravasation can lead to increased image noise and attenuation of defects developed during peak stress (29). Extravasation during renal scintigraphy studies, such as calculation of glomerular filtration rate (GFR), can invalidate the findings due to delayed uptake and washout (30). And in brain imaging, extravasation can reduce uptake and thus feature identification (31).

To enable the best possible care, nuclear medicine imaging studies must present information that is as clear and unambiguous as possible. Extravasation is one factor that reduces clarity and complicates image interpretation. For that reason, monitoring of injections in both general nuclear medicine and PET/CT has become a routine practice at our institution. By identifying and characterizing extravasations, we are able to proactively estimate the impact on image reliability and also better communicate with the patient about their care. On several occasions, we have determined that a patient would be better served by re-imaging rather than using the images from an extravasated injection. The decision to do so, however, is based on several factors.

## 2. The decision-making process

When deciding whether to repeat a study, our primary consideration is whether the image satisfies the diagnostic needs of the referring physician. We strive to provide complete and accurate reports without equivocation or disclaimers. If specific clinical questions cannot be answered due to technical or quality-related issues, the study may have to be repeated.

**Figure 1:**
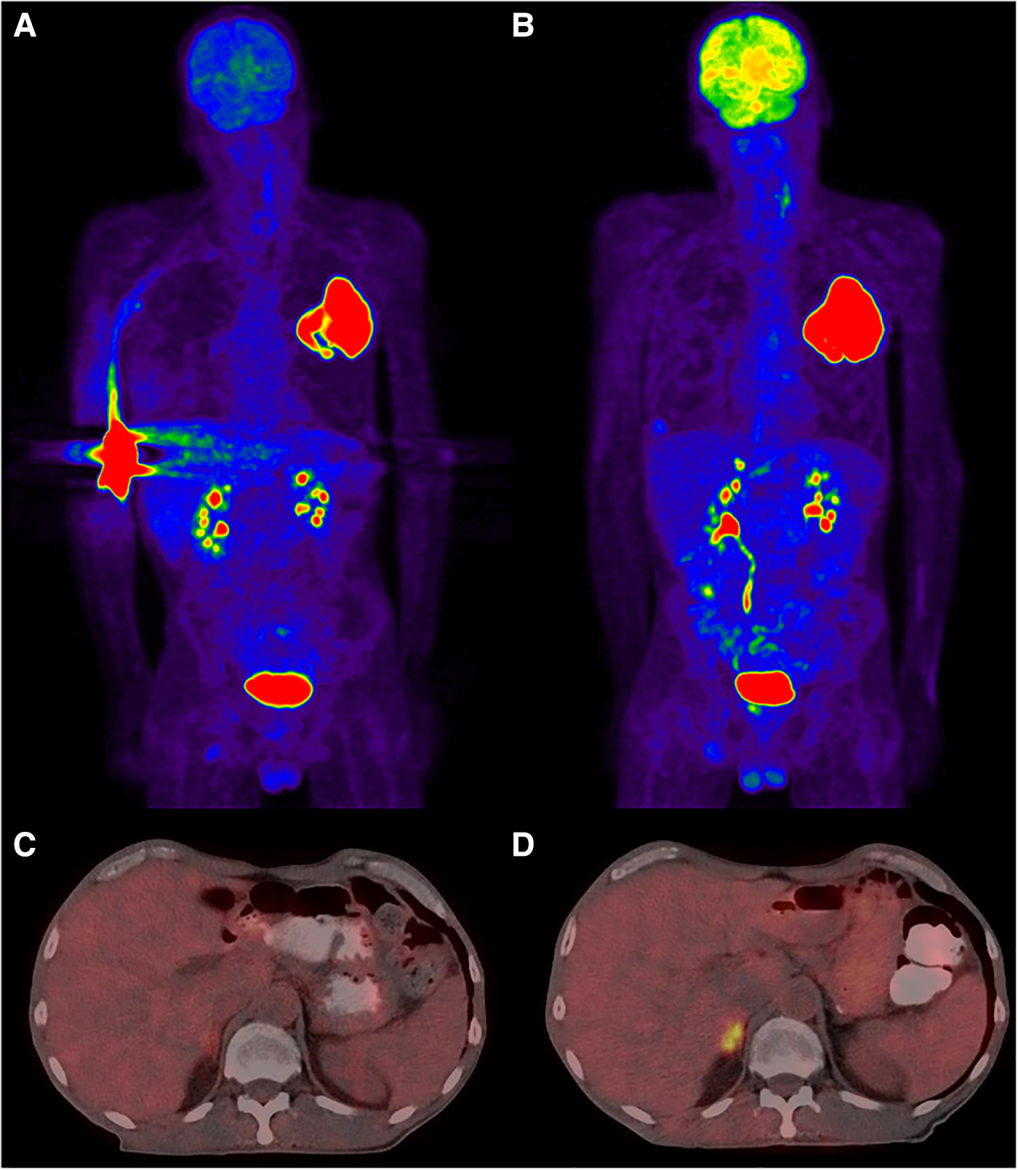
PET/CT images demonstrating the implications of a large extravasation. Subparts (**A**) and (**C**) correspond to the extravasated imaging, (**B**) and (**D**) are from reimaging three days later with no extravasation. Subpart (**A**): maximum intensity projection (MIP) showing extravasation in the right antecubital and a large mass in the upper left lung. Subpart (**C**): slice at the renal level indicating no obvious occult uptake. Subpart (**B**): MIP view now with an additional lesion evident in the adrenals. Subpart (**D**): slice from the renal level showing increased focal uptake within the adrenals.

It is true that some diagnostic studies are minimally affected by extravasation. For example, when investigating a fever of unknown origin, most causes of pathologic uptake will be diagnostic and need not have accurate SUV measurements. The one exception might be in the setting of tumor fever in an undiagnosed or occult cancer. For other studies, though, the impact on patient care can be significant. In a previously published report from our institution (27), PET imaging revealed a single lung mass with no nodal involvement or metastases (Figures 1A, C). Initial disease staging was T3N0M0. However, because of significant extravasation, we questioned the reliability of the findings. The patient was reimaged three days later, and additional disease was evident in the right adrenal (Figures 1B, D). Disease staging was revised to T3N0M1. Extravasation affected the images such that they did not accurately answer the clinical questions being asked, and our decision to reimage resulted in a significant change to disease management for the patient. Based on revised staging, the patient was no longer a candidate for the previously planned surgical debulking and adjuvant therapy. The patient chose to enter hospice care and passed away not long thereafter.

In another example^1^, a patient was referred for staging prior to external beam radiation therapy. Four lesions were evident in the image, but we also noted significant extravasation. We decided to repeat the study after considering the intent of the referring physician and the potential ramifications of an inaccurate study; we scheduled the patient to be reimaged 5 days later. Based on the second PET scan, metabolic Tumor Volume (MTV) calculations from the original were found to have been understated by 46% on average. Use of the inaccurate MTVs could have resulted in inadequate radiation therapy for this patient.

Secondary factors may also affect the decision to reimage. These factors include additional patient radiation dose, patient burden, and administrative constraints.

Nuclear medicine procedures necessarily expose the patient to radiation dose. When deciding to repeat a portion of the procedure, we consider whether the benefit outweighs the risk associated with the additional radiation dose. It may be appropriate to document the risk-benefit analysis—particularly for cases involving children or breastfeeding mothers. This practice is consistent with the principles of “As Low As Reasonably Achievable” (ALARA) and with the recent industry focus on reduction of radiation doses in nuclear medicine^2^^,^^3^.

Burden on the patient is another factor that we consider. For many patients, difficulty with transportation, childcare, time away from work, and other factors can be significant barriers to care (32). When these factors impact a patient, they may be less willing to attend a rescheduled procedure.

Administrative constraints, including the cost to reimage compromised procedures, can also play an important role in the decision-making process. Our institution accepts that it sometimes may be necessary to repeat a procedure, and we reimage with no additional cost to the patient. Our ability to do this, though, is reliant on past work that we have done to reduce the overall frequency of extravasation (33). By using the “Define, Measure, Analyze, Improve, Control” process improvement technique over a time period of 25 weeks, we have reduced the incidence of extravasation in PET/CT by over 78% (from 13.3% to 2.9%, *P* < 0.001) (34). Since improving the quality of our radiopharmaceutical injections, we typically consider reimaging only a few patients per year.

For example, in 2022, we monitored 2,060 PET/CT injections. Of those, we determined 2,001 (97.1%) to be ideal, 52 (2.5%) to be negligibly extravasated, and 7 (0.3%) to be moderately or severely extravasated. Cases of negligible extravasation can be quickly confirmed to have no impact on the patient or imaging procedure. For more severe examples, a simple process of characterization can be used to assess not only the need for reimaging, but also the potential radiological affects to the patient (9, 10). Based on the criteria discussed above, only one case warranted reimaging. Our institution accepts occasional reimaging as one of the many costs of ensuring that we provide high quality images and reports, but we continue to work toward minimizing the frequency of extravasation.

Each of the factors above are important parts of the overall decision to reimage with the primary goal being to provide accurate and definitive answers to the clinical questions being asked. Sometimes that goal can be achieved using diagnostic images that are compromised, but in cases where the reading physician's interpretation is ambiguous, the secondary decision factors are of limited importance.

## 3. Patient communication

Several authors describe the importance of procedure-specific information, including information about risks, in the management of patient anxiety (35–38). Less has been written, though, about the process of discussing problems during patient care. Some hold a belief that communicating with patients about problems should be avoided. For example, in a recent statement concerning extravasations, the Health Physics Society said, “Additional documentation of the survey and measurement of infiltration would need to be included in the medical record, adding further to additional burden and raising the issue of needlessly alarming patients” (39). Likewise, in an official response to the a proposal to consider extravasations as reportable medical events, one commenter said, “Classifying extravasation as medical even[ts] would mean that it must be communicated to patient. Many patients who already have to cope with cancer or other serious diseases would have to deal with this additional piece of information, no matter how trivial it might be” (40). In contrast, we do not agree that communicating with patients about extravasation is alarming, needless, or trivial.

At our institution, we prospectively monitor the administration site for extravasation with the latest technology and we explain to patients the importance of the quality of the administration. When we are alerted that excess radiotracer is present near the injection site as compared to the levels in the opposite arm, we include the injection site in the imaging field-of-view. After confirming extravasation, our standard procedure is for the nuclear medicine technologists to talk with the patient and explain what happened. Because of the latent effects of ionizing radiation and because patients may not realize they have been extravasated, our technologists discuss possible localized effects (e.g., erythema, pain) that may be experienced. Then, based on a determination by the radiology physician, our technologists work with the patients to schedule a repeat imaging scan.

In our experience, the overwhelming response is that patients appreciate that we prioritize the quality of the study, and they are willing to repeat procedures when asked. It is not uncommon for patients to be well educated about their care and the procedures they undergo. Access to information from the internet and their own electronic medical records has increased both patient involvement and their appetite for substantive communication. Furthermore, we keep the patient's referring physician informed so that they can continue to make appropriate care judgements.

Recently, the U.S. Nuclear Regulatory Commission (NRC) accepted a petition for rulemaking that certain extravasations be reported. The Commission has initiated rulemaking, but suggested that only those extravasations which result in tissue damage as confirmed by a physician authorized-user or that providers suspect could result in tissue damage should be reportable events (41). Presumably, all other radiopharmaceutical extravasations would be exempted from reporting and there would be no requirement to notify patients or their referring physicians. We worry that this recommendation may encourage providers to accept poor injection quality rather than work to improve their training and techniques. Given the impact that extravasation can have on the quantification and interpretation of nuclear medicine studies, it is worthwhile to reduce the frequency of all extravasations, not only those that could cause serious injury. We believe patients, and their referring physicians, deserve to know when a technical or otherwise procedural error has occurred even if no injury is identified. Chamberlain et. al., argue that it is in fact best practice to communicate fully, even in the case of apparently harmless errors: “Full disclosure of minimal-harm or near-miss errors strengthens the patient-surgeon relationship, cultivates an open atmosphere of dialogue, and facilitates patient participation in medical decision making” (42).

## 4. Conclusion

Radiopharmaceutical extravasation can result in serious consequences to the qualitative and quantitative value of nuclear medicine imaging studies. There may also be follow-on effects to the patient's overall care and wellbeing. To provide high quality service, nuclear medicine departments as well as the interpreting radiologist physicians should be cognizant of the possible need to repeat studies which have been affected by an extravasation. The decision to reimage is based on several factors, but chief among them should always be the clinical intent of the procedure. Additionally, patients expect and deserve to be told when a medical procedure does not go according to plan. Providers should be prepared to communicate the circumstances, explain the possible ramifications, and ensure appropriate actions are taken.

## Data Availability

The original contributions presented in the study are included in the article/Supplementary Material, further inquiries can be directed to the corresponding author.

## Footnotes

^1^Unpublished data.

^2^Image Wisely, https://www.imagewisely.org/, accessed March 24, 2023.

^3^Image Gently, http://www.imagegently.org/, accessed March 24, 2023.
